# Hot Deformation Behavior, Processing Maps and Microstructural Evolution of the Mg-2.5Nd-0.5Zn-0.5Zr Alloy

**DOI:** 10.3390/ma15051745

**Published:** 2022-02-25

**Authors:** Junfei Ma, Songhui Wang, Jianlei Yang, Wencong Zhang, Wenzhen Chen, Guorong Cui, Guannan Chu

**Affiliations:** 1 School of Materials Science and Engineering, Harbin Institute of Technology, Harbin 150001, China; majingxuan0810@163.com (J.M.); jlyang@hit.edu.cn (J.Y.); zhangwencong@hit.edu.cn (W.Z.); nclwens@hit.edu.cn (W.C.); cuiguorong2010@126.com (G.C.); chuguannan@hit.edu.cn (G.C.); 2 Weihai Lightweight Materials and Forming Engineering Research Center, Weihai 264209, China

**Keywords:** Mg-2.5Nd-0.5Zn-0.5Zr alloy, hot deformation, microstructure, constitutive equations

## Abstract

Isothermal hot compression experiments were conducted on Mg-2.5Nd-0.5Zn-0.5Zr alloy to investigate hot deformation behavior at the temperature range of 573–773 K and the strain rate range of 0.001 s^−1^–10 s^−1^ using a Gleeble-3500D thermomechanical simulator. The results showed that the rheological curve showed a typical work hardening stage, and there were three different stages: work hardening, transition and steady state. A strain compensation constitutive model was established to predict the flow stress of the Mg-2.5Nd-0.5Zn-0.5Zr alloy, and the results proved that it had high predictability. The main deformation mechanism of the Mg-2.5Nd-0.5Zn-0.5Zr alloy was dislocation climbing. The processing maps were established to distinguish the unstable region from the working region. The maps showed that the instability generally occurred at high strain rates and low temperatures, and the common forms of instability were cracking and flow localization. The optimum machining range of the alloy was determined to be 592–773 K and 0.001–0.217 s^−1^. With the increase in deformation temperature, the grain size of the alloy grew slowly at the 573–673 K temperature range and rapidly at the 673–773 K temperature range.

## 1. Introduction

Mg alloys have the benefits of low density, high stiffness, high specific strength and machinability [1]. They have been widely used in aerospace, automotive, electronics, medical devices and other industries [2,3,4]. The mechanical and structural properties of Mg alloys and their response to stresses are currently a hot topic [5,6,7]. Many studies have shown that rare earth (RE) elements could significantly strengthen Mg alloys [8,9,10,11,12]. Although adding a large number of RE elements can effectively improve the mechanical properties of an alloy, the cost increases greatly, which limits the wide application of Mg alloys with high RE content [13,14]. Therefore, the rapid development of high-performance low-RE content Mg alloys is urgent. 

Mg-Nd-Zn-Zr alloys are favored by many researchers for their excellent mechanical properties and low RE content [15,16,17,18]. However, the limited thermoplastic processing conditions of Mg-RE alloys restrict their further application [19]. Therefore, a comprehensive understanding of the thermal deformation behavior of Mg-Nd-Zn-Zr alloys was necessary for the development and engineering application of low RE content Mg alloys. Up to now, the research on Mg-Nd-Zn-Zr alloys mainly focused on the effect of casting and heat treatment on the microstructure and properties of the alloy [11,14,20]. Cast alloys generally have many defects (e.g., coarse structure, low strength and poor plasticity, etc.) [11,14,18,20,21]. Hot extrusion was shown to be an effective method to refine the microstructure, eliminate casting defects and improve the properties of alloys, which can significantly improve its hot formability [21]. However, there was little existing research on the hot working ability of the extruded Mg-Nd-Zn-Zr alloys. Therefore, it was of great significance to study the thermal deformation behavior and microstructure evolution of the extruded Mg-Nd-Zn-Zr alloy, and to optimize its thermal processing parameters using the processing map. 

It is well known that constitutive equation analysis has become an effective method to present the thermal deformation behavior of metal materials [22]. The flow behavior of materials during thermal deformation is complicated and is mainly affected by deformation parameters (e.g., strain rate, deformation temperature and deformation degree, etc.). Hence, a suitable constitutive model should consider the coupling effects of multiple factors simultaneously. As an improved form of the hyperbolic sinusoidal Arrhenius model, the strain-compensated Arrhenius equation can meet the above requirements and has been diffusely used in studies of thermal deformation of metal materials (e.g., magnesium alloy [23,24], aluminum alloy [25,26], titanium alloy [27,28], etc.). For example, Hu et al. [29] constructed the strain-compensated Arrhenius constitutive model for Mg-8.5Gd-4.5Y-0.8Zn-0.4Zr alloy. The predicted values were in good agreement with the experimental results. Zhang et al. [30] investigated the deformation behavior of homogenized Mg-5.25Gd-2.73Y-0.51Zr alloy, and a constitutive equation of strain compensation was proposed. The experimental results were in good agreement with the predicted results. Thus far, the literature has showed that there are few existing studies on the thermal deformation behavior of Mg-Nd-Zn-Zr alloys using the strain-compensated Arrhenius model, especially the studies on the strain-compensated constitutive equation of extruded Mg-Nd-Zn-Zr alloys. 

The dynamic material model (DMM) of Prasad based on processing maps is a powerful tool for optimizing thermal processing and controlling the microstructure of a variety of alloys [23,25,26,27]. For instance, Xia et al. [23] investigated the hot workability of Mg-6.85Gd-4.52Y-1.15Nd-0.55Zr alloy by compression tests and established processing maps. The suitable hot working condition for the alloy was determined to be 773 K and 0.05 s^−1^. Zheng et al. [31] studied the hot tensile deformation behavior of extruded Mg-2.5Nd-0.2Zn-0.1Zr alloy at a temperature range of 200–400 °C and a strain rate of 0.001–1 s^−1^, and established the hot working diagram. The optimum hot working condition temperature was 380 °C and the strain rate was 0.006 s^−1^.

In this paper, the deformation behavior of the extruded Mg-2.5Nd-0.5Zn-0.5Zr alloy was investigated using the Gleeble-3500D thermal simulator. A strain-compensated Arrhenius constitutive equation was established to predict the flow stress. A DMM-based processing chart was constructed, which provided important guidance for optimizing thermal deformation parameters. In addition, the evolution of the microstructure of the alloy was analyzed.

## 2. Material and Experimental Procedures

**Figure 1:**
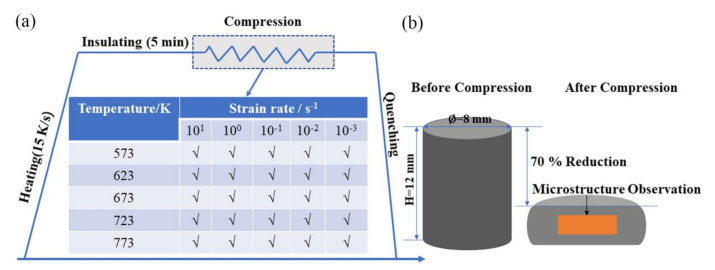
Diagram of the isothermal compression process. (**a**) experimental process; (**b**) EBSD sample.

The initial material (provided by Guizhou Anji Nonferrous Casting Co., Ltd., Guizhou, China) was an Mg-2.5Nd-0.5Zn-0.5Zr (wt%) extrusion bar with a diameter of 25 mm. The actual chemical composition (Mg-2.58Nd-0.54Zn-0.54Zr, wt%) was examined by X-ray fluorescence spectrometer (ZSX-100e, Tokyo, Japan). The compression sample was processed along the extrusion direction (ED), and the cylindrical sample was Φ8 × 12 mm. Firstly, the surfaces were polished smooth with 400#, 800#, 1200# and 1500# sandpaper, and then the samples were cleaned by ultrasonic shock for 5 mins in acetone and alcohol solution, in that order. The isothermal compression experiment was conducted using the Gleeble-3500D thermal simulator. Experimental parameters are shown in Figure 1. All samples were compressed to the true strain of 0.7, and then the sample were taken out for quenching to retain the high-temperature deformation structure. Note that the graphite papers were placed at the ends of the samples to avoid the experimental error caused by friction before the experiment.

With the inhomogeneous deformation of the sample taken into consideration, the microstructure was observed in the center area of the longitudinal section, as shown in the brown area in Figure 1b. The microstructure analysis was undertaken by electron backscatter diffraction (EBSD, MERLIN Compact, Oberkochen, Germany). The EBSD samples were first prepared by mechanical polishing, then electropolishing (5:3 C_2_H_5_OH and H_3_PO_4_ electrolyte, electropolishing for 2 min at 0.5 A and 5 min at 0.25 A).

## 3. Results and Discussion

### 3.1. Stress–Strain Curves

**Figure 2:**
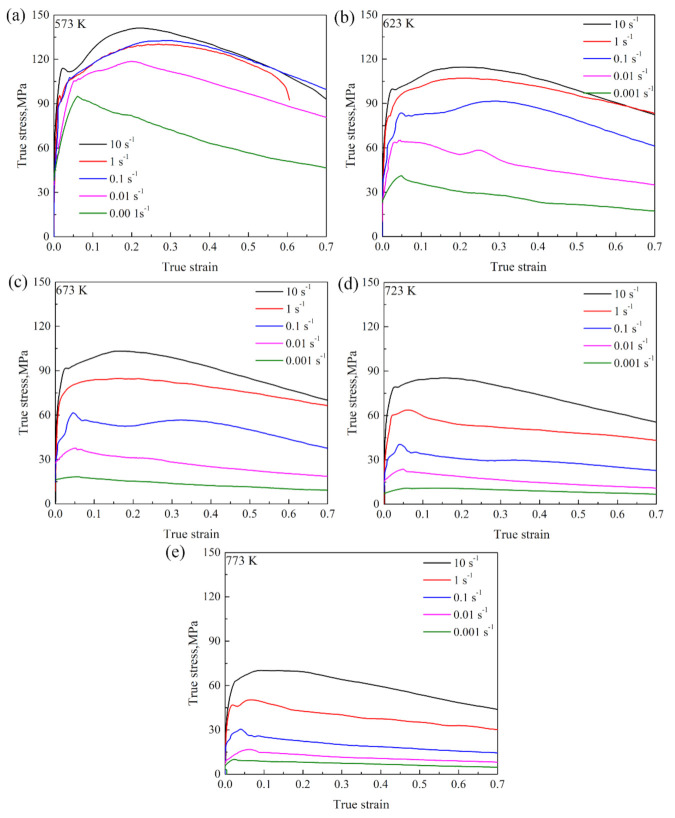
True stress–strain curves of the Mg-2.5Nd-0.5Zn-0.5Zr alloy at various hot deformation temperatures: (**a**) 573 K, (**b**) 623 K, (**c**) 673 K, (**d**) 723 K, (**e**) 773 K.

Figure 2 exhibits the true stress-strain curves of the Mg-2.5Nd-0.5Zn-0.5Zr alloy under different deformation parameters. It reflects the process of work hardening and dynamic recrystallization (DRX) during deformation. Each stress–strain curve includes the work-hardening stage, dynamic-softening stage and dynamic-equilibrium stage. At the initial stage of deformation, with the accumulation of compression, the flow stress increased sharply to the maximum stress value. As the hot compression continued, varying degrees of softening arose so that the flow stress gradually decreased and finally reached the equilibrium state. Take the deformation condition of 573 K/0.001 s^−1^ in Figure 2a as an example: when the compression strain was between 0 and 0.04, the flow stress of the Mg-2.5Nd-0.5Zn-0.5Zr alloy increased rapidly from 0 MPa to ~90 MPa, which was attributed to the work-hardening phenomenon caused by the proliferation of dislocation, delivery entanglement and dislocation movement obstruction [32,33,34,35]. With the increase in strain, dynamic recovery (DRV) increased and work hardening decreased. However, DRV could not completely offset the distortion energy caused by work hardening, and the increased rate of flow stress decreased. DRX occurred when the critical strain reached 0.06. DRX significantly reduced dislocation density, and the flow stress reached its peak at 103.4 MPa. After further accumulation of strain variables, the softening effect of DRV and DRX gradually exceeded the work-hardening effect, and the flow stress showed a downward trend. When the strain reached about 0.5, work hardening and dynamic softening reached a dynamic equilibrium, and the flow stress tended to equilibrium.

**Figure 3:**
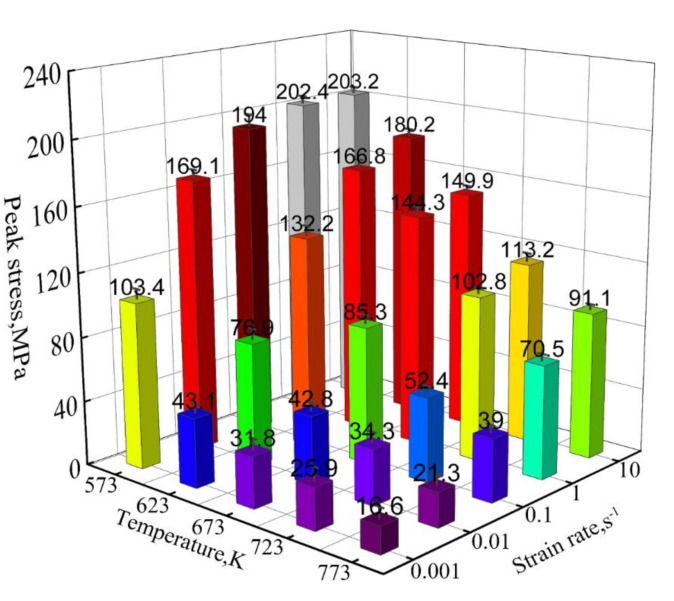
Peak flow stress of the Mg-2.5Nd-0.5Zn-0.5Zr alloy at different deformation conditions.

Figure 3 presents the peak stress of the Mg-2.5Nd-0.5Zn-0.5Zr alloy under different deformation conditions. At the same strain rate, the peak stress of the Mg-2.5Nd-0.5Zn-0.5Zr alloy decreased gradually with the increase in deformation temperature. This was mainly because the atomic kinetic energy of the alloy increased gradually with the increasing deformation temperature, which led to a decrease in interatomic bonding ability and a decrease in the deformation resistance of the alloy [2]. Meanwhile, the thermal activation energy of the Mg-2.5Nd-0.5Zn-0.5Zr alloy decreased with the increase in temperature, which led to an increase in actual dislocations and slip systems in the microstructure, and the dynamic softening effect was obvious. At the same deformation temperature, the peak stress of the alloy decreased with the decrease in strain rate, which could be attributed to the fact that the dislocation generated during the low strain rate compression process had had enough time to start, the dislocation entanglement phenomenon was weakened and the dynamic softening effect was significant [30]. Meanwhile, the lower deformation rate could have improved the stress concentration caused by uneven grain deformation. The increase in temperature and the decrease in strain rate also led to the nonlinear decrease in peak stress. The maximum peak stress was 203.2 MPa at 573 K 10 s^−1^ and the minimum peak stress was 16.6 MPa at 773 K 0.001 s^−1^.

### 3.2. Constitutive Model

#### 3.2.1. Constitutive Equations

An Arrhenius-type equation was used to characterize the relationship between the hot-deformation parameters [22,23,24,25,26,27,28,29,30,31], as follows:

(1) $$\dot{\varepsilon }=\mathrm{AF}\left(\sigma \right)\exp\left(-\frac{Q}{\mathrm{RT}}\right)$$

(2) $$F\left(\sigma \right)=\left\{\begin{array}{c} \sigma^{n_{1}}\left(\alpha \sigma <0.8\right)\;\;\\ \exp\left(\beta \sigma \right)(\alpha \sigma >0.8)\\ {\left[\sinh\left(\alpha \sigma \right)\right]}^{n}\left(\mathrm{For}\;\mathrm{all}\right) \end{array}\;\right.$$

where $\dot{\varepsilon }$ was the strain rate (s^−1^), σ was the flow stress (MPa), Q was the apparent deformation-activation energy (kJ/mol), T was the deformation temperature (K), R was the gas constant (R = 8.314 J/mol K), and A, $n_{1}$, α, β were material constants. In addition, α=βn1.

(3) $$\ln\dot{\varepsilon }=\mathrm{lnA}+n_{1}\ln\sigma -\frac{Q}{\mathrm{RT}}$$

(4) $$\ln\dot{\varepsilon }=\mathrm{lnA}+\beta \sigma -\frac{Q}{\mathrm{RT}}$$

(5) $$\ln\dot{\varepsilon }=\mathrm{lnA}+\mathrm{nln}\left[\sinh\left(\alpha \sigma \right)\right]-\frac{Q}{\mathrm{RT}}$$

From Equations (6)–(9), the parameters $n_{1}$, β, n and Q could be expressed as:

(6) $$n_{1}={\left(\frac{\partial \ln\dot{\varepsilon }}{\partial \ln\sigma }\right)}_{T}$$

(7) $$\beta ={\left(\frac{\partial \ln\dot{\varepsilon }}{\partial \sigma }\right)}_{T}$$

(8) $$n={\left[\frac{\partial \ln\dot{\varepsilon }}{\partial \mathrm{lnsinh}\left(\alpha \sigma \right)}\right]}_{T}$$

(9) Q=nR[∂lnsinhασ∂1T]T

The relationship between strain rate ($\dot{\varepsilon }$) and deformation temperature (T) was expressed as [22]:

(10) $$Z=\dot{\varepsilon }\exp\left(-\frac{Q}{\mathrm{RT}}\right)=A{\left[\sinh\left(\alpha \sigma \right)\right]}^{n}$$

Linearizing Equation (11) by logarithmic processing:

(11) $$\mathrm{lnZ}=\mathrm{lnA}+n\left[\sinh\left(\alpha \sigma \right)\right]$$

**Figure 4:**
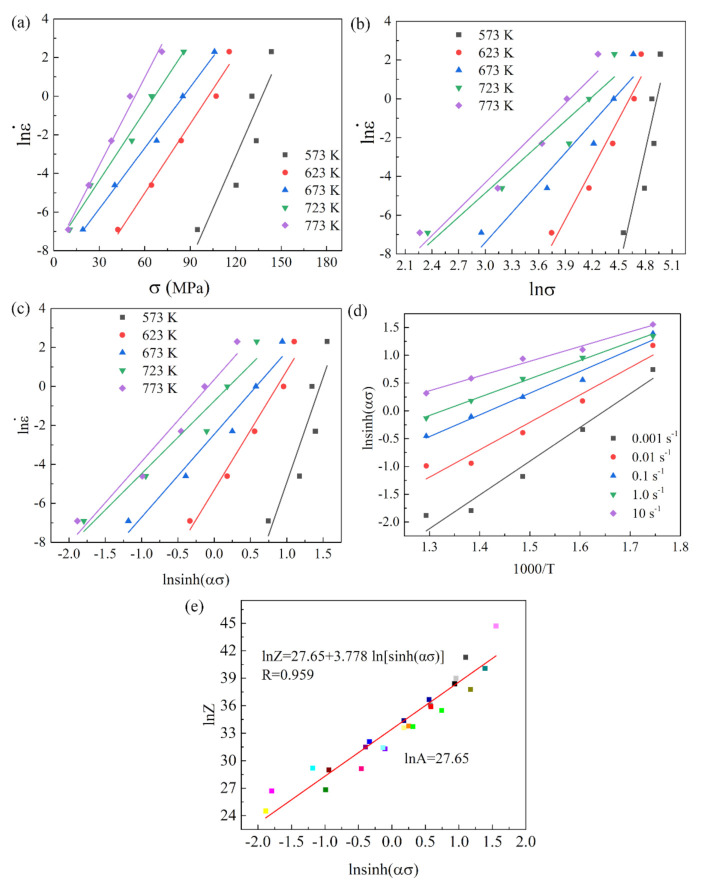
Linear relationships of (**a**) σ- ln$\dot{\varepsilon }$, (**b**) lnσ- ln$\dot{\varepsilon }$, (**c**) ln[sinh(ασ)]-ln$\dot{\varepsilon }$, (**d**) 1000/T- ln[sinh(ασ)] and (**e**) lnZ-ln[sinh(ασ)].

In this study, the detailed steps to determine the material constants were illustrated by taking a strain of 0.4 as an example. In Figure 4, based on Equations (6)–(8), the values of $n_{1}$, β and n were calculated from the slopes of $\ln\sigma -\ln\dot{\varepsilon }$, $\sigma -\ln\dot{\varepsilon }$, and $\ln\left[\sinh\left(\alpha \sigma \right)\right]-\ln\dot{\varepsilon }$, Q and lnA. The β, α, n, n_1_, Q, and lnA were 0.121014, 0.021944, 3.778, 5.5147, 171.929 kJ/mol and 27.65, respectively. 

The flow stress can be expressed as:

(12) $$\sigma =\frac{1}{\alpha }\ln\{{\left(Z/A\right)}^{1/n}+{\left[{\left(Z/A\right)}^{2/n}+1\right]}^{1/2}\}$$

Hence, the flow stress of the Mg-2.5Nd-0.5Zn-0.5Zr alloy when the strain was 0.4 can be expressed as:

(13) $$\sigma =45.5705\ln\left\{{\left(Z/e^{27.65}\right)}^{\frac{1}{3.778}}+{\left[{\left(Z/e^{27.65}\right)}^{\frac{2}{3.778}}+1\right]}^{\frac{1}{2}}\right\}$$

#### 3.2.2. Strain Compensation Analysis

**Figure 5:**
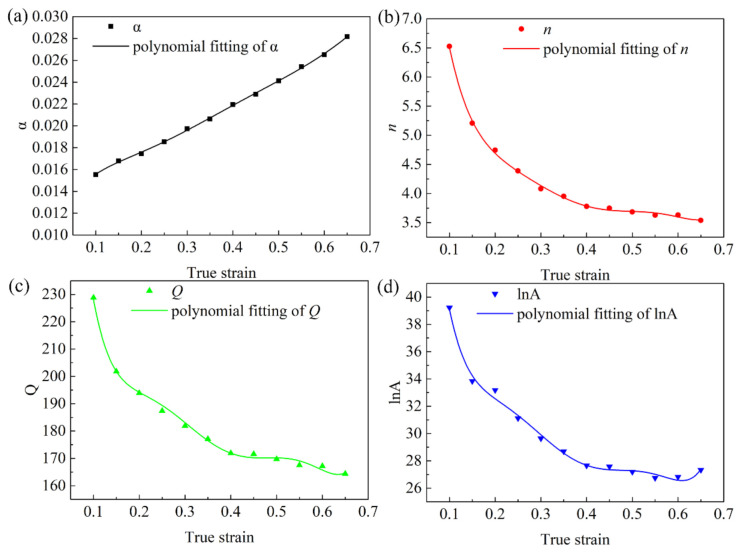
Plots of the compensation functions of different material parameters: (**a**) a, (**b**) n, (**c**) Q, and (**d**) lnA.

It can be seen from Figure 2 that the true strain variable (ε) also had a great effect on the flow stress of the Mg-2.5Nd-0.5Zn-0.5Zr alloy. However, in previous studies [3,23,30], a single strain was usually used to establish a simple constitutive equation, and strain-factor parameters were not added in Equation (13). The result showed that the calculated thermal Q could not reflect the real activation energy in the actual deformation process. Therefore, based on the true stress–strain curve of the Mg-2.5Nd-0.5Zn-0.5Zr alloy in Figure 2, the material constants, corresponding to the true strain from 0.1 to 0.65, were calculated using the method of ε=0.4 with 0.05 intervals. Then, a series of α, n, Q and lnA values were polynomially fitted with the true strain, as shown in Equation (14). The function relation between the material constants α, n, Q, lnA and the true strain was established. Figure 5 shows the result of sixth-degree polynomial fitting, and the polynomial coefficients are shown in Table 1.

(14) $$\begin{array}{c} \alpha =B_{0}+B_{1}\varepsilon^{1}+B_{2}\varepsilon^{2}+B_{3}\varepsilon^{3}+B_{4}\varepsilon^{4}\ldots +B_{m}\varepsilon^{m}\\ n=C+C_{1}\varepsilon^{1}+C_{2}\varepsilon^{2}+C_{3}\varepsilon^{3}+C_{4}\varepsilon^{4}\ldots +C_{m}\varepsilon^{m}\\ Q=D_{0}+D_{1}\varepsilon^{1}+D_{2}\varepsilon^{2}+D_{3}\varepsilon^{3}+D_{4}\varepsilon^{4}\ldots +D_{m}\varepsilon^{m}\\ \mathrm{lnA}=E_{0}+E_{1}\varepsilon^{1}+E_{2}\varepsilon^{2}+E_{3}\varepsilon^{3}+E_{4}\varepsilon^{4}\ldots +E_{m}\varepsilon^{m} \end{array}$$

The constant equation with the strain factor (ɛ) was coupled to the constructed constitutive equation. Therefore, the hyperbolic sinusoidal Arrhenius constitutive equation of the Mg-2.5Nd-0.5Zn-0.5Zr alloy was obtained at 573–773 K and a strain rate of 10.0–0.001 s^−1^, as shown in Equation (15).

(15) $$\begin{array}{c} \sigma =\frac{1}{\alpha }\ln\{{\left(Z/A\right)}^{1/n}+{\left\{{\left(Z/A\right)}^{2/n}+1\right]}^{1/2}\}\\ \alpha \left(\varepsilon \right)\;=\;0.01\;+\;0.09\varepsilon^{1}-\;0.65\varepsilon^{2}+\;2.54\varepsilon^{3}-\;5.09\varepsilon^{4}+\;5.03\varepsilon^{5}-\;1.93\varepsilon^{6}\\ n\left(\varepsilon \right)\;=\;15.56\;-\;170.07\varepsilon^{1}+\;1127.68\varepsilon^{2}-\;4034.49\varepsilon^{3}+\;7904.61\varepsilon^{4}-\;7960.32\varepsilon^{5}+\;3217.49\varepsilon^{6}\\ Q\left(\varepsilon \right)\;=\;507.57\;-\;5781.23\varepsilon^{1}+\;43757.45\varepsilon^{2}-\;170718.30\varepsilon^{3}+\;354866.01\varepsilon^{4}-\;373319.75\varepsilon^{5}+\;156209.15\varepsilon^{6}\\ \mathrm{InA}\left(\varepsilon \right)\;=\;88.95\;-\;1033.31\varepsilon^{1}+\;7893.32\varepsilon^{2}-\;31410.53\varepsilon^{3}+\;66790.89\varepsilon^{4}-\;71967.43\varepsilon^{5}+\;30880.84\varepsilon^{6} \end{array}$$

#### 3.2.3. Deformation Mechanism

**Figure 6:**
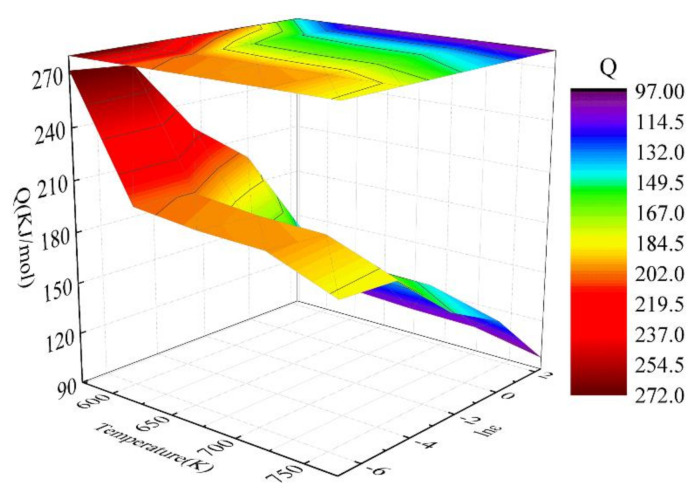
3D activation energy map of the Mg-2.5Nd-0.5Zn-0.5Zr alloy at a strain of 0.4.

The deformation mechanism of the alloy was mainly affected by the stress exponent (n) and Q in the constitutive model. The change of n value was related to the movement of crystal defects during deformation. In Table 2, different n values correspond to different deformation mechanisms. In particular, according to Equations (8) and (9), the corresponding Q and n under strain (ε = 0.4) were calculated. The calculated results are presented in Table 3. Figure 6 shows the three-dimensional activation energy distribution of the Mg-2.5Nd-0.5Zn-0.5Zr alloy at a strain of 0.4. The Q decreased with the increase in strain rate and deformation temperature. The Q was in the range of 97.1-271.9 kJ/mol. The grain boundary diffusion activation energy (Q_GB_) of pure Mg was 82–105 kJ/mol, and the lattice self-diffusion activation energy (Q_L_) of pure Mg was 135 kJ/mol [36,37]. According to Figure 5b and Table 3, when the strain rate was 10 s^−1^ and the deformation temperature was 723–773 K (the black area in Table 3), the Q of the alloy was close to the Q_GB_ of pure Mg, and the deformation mechanism of the alloy was dislocation climbing controlled by grain boundary diffusion. The Q of the alloy was greater than the Q _L_ and less than the Q_GB_ of pure Mg at strain rates of 1–10 s^−1^ and 623–773 K (the blue area in Table 3), and the deformation mechanism of the alloy was a dislocation climbing mechanism. The Q of the alloy was greater than the Q_L_ of pure Mg at strain rates of 0.001–10 s^−1^ and 573–773 K (the red area in Table 3); these results indicated that there were other mechanisms besides lattice diffusion that led to the increase in the Q of thermal deformation [38]. The deformation was controlled by the joint mechanism of second-phase pinning and dislocation, and the source of the pinning may be related to solute atoms and dynamic precipitates [39]. Therefore, higher energy was required to induce dislocation climbing. Similarly, this phenomenon has been reported in research be Lei et al. [26].

#### 3.2.4. Assessment of the Constitutive Model

To further verify the applicability of the true strain-coupled high-temperature constitutive equation established in the above section, the correlation coefficient R, square root error (RMSE) and average relative error (AARE) were used for comprehensive evaluation [4,26,28]. Assessment of the predictability of the devised model was performed by the RMSE, R, and AARE presented in Equations (16)–(18), respectively [26,28].

(16) $$\mathrm{RMSE}=\sqrt{\sum_{i=1}^{N}{\left(E_{i}-P_{i}\right)}^{2}/N}$$

(17) $$R=\frac{\sum_{i=1}^{N}\left(E_{i}-\bar{E}\right)\left(P_{i}-\bar{P}\right)}{\sqrt{\sum_{i=1}^{N}(E_{i}-{\bar{E})}^{2}{\left(P_{i}-\bar{P}\right)}^{2}}}\;$$

(18) $$\mathrm{AARE}=\frac{1}{N}=\sum_{i=1}^{N}\left|\frac{E_{i}-P_{i}}{E_{i}}\right|$$

**Figure 7:**
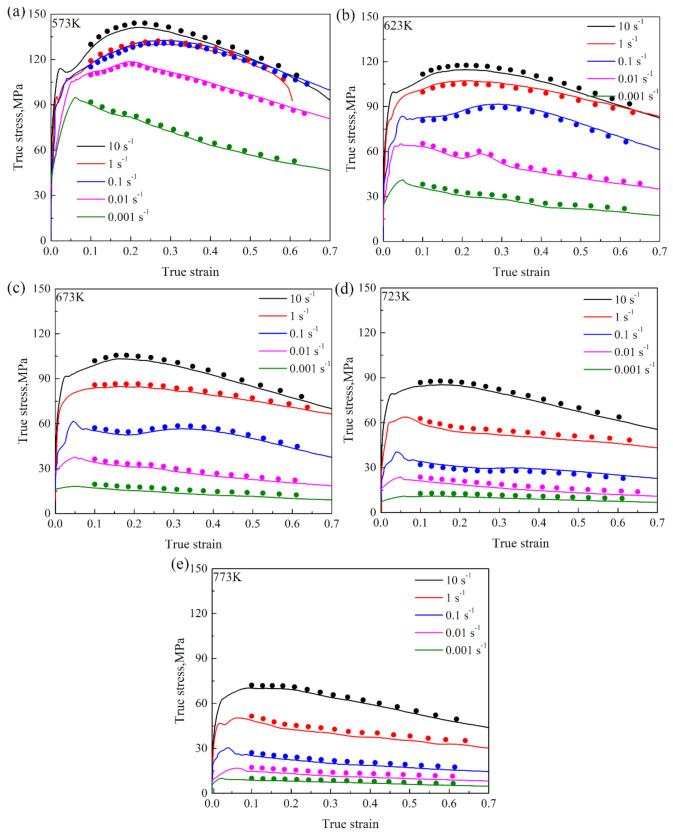
Experimental (solid lines) and predicted (scatter plot) flow stress of the alloy at (**a**) 573 K, (**b**) 623 K, (**c**) 673 K, (**d**) 723 K and (**e**) 773 K.

**Figure 8:**
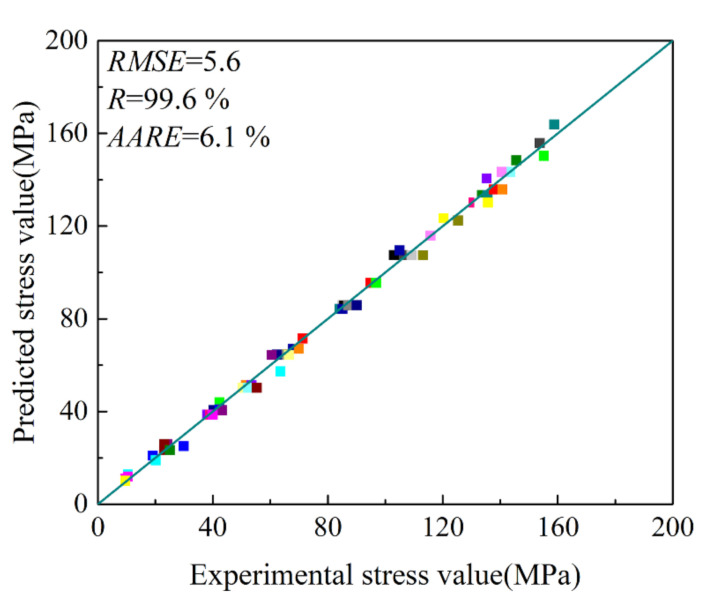
Correlation between the experimental and predicted flow stress.

From Equations (16)–(18), E was the experimental flow stress, P was the predicted flow stress, and i stands for the ordinal. N was the number of data sets for statistics. As shown in Figure 7, the deviation between the experimental flow stress (solid line) and the predicted value (scatter diagram) of the established high-temperature constitutive model was small, indicating that the prediction of flow stress by the constitutive model was relatively accurate. In Figure 8, the RMSE of the model was 5.6, the predictability of the established high-temperature constitutive model was 99.6% and the AARE was only 6.1%, which verified the high accuracy of the constitutive model. It was shown that the constitutive model could accurately describe the relationship between the flow stress, deformation temperature and strain rate of the Mg-2.5Nd-0.5Zn-0.5Zr alloy studied.

### 3.3. Processing Map

Prasad et al. [26,27] developed a processing map based on DMM. In this model, the total power dissipated (P) consisted of two parts. One was the power dissipated by plastic deformation, which was expressed by G content, and the other was the power dissipated by microstructure evolution (e.g., DRV, DRX and phase transition, etc.), represented by J content. Therefore, P was expressed by the following equation:

(19) $$P=G+J=\sigma \dot{\varepsilon }=\int_{0}^{\varepsilon }\sigma d\dot{\varepsilon }+\int_{0}^{\sigma }\dot{\varepsilon }d\sigma$$

For arbitrary strain rate and deformation temperature, the power distribution between J and G was described as follows [29,31]:

(20) $${\left(\frac{\partial J}{\partial G}\right)}_{T,\dot{\varepsilon }}={\left(\frac{\partial \ln\sigma }{\partial \ln\dot{\varepsilon }}\right)}_{T,\dot{\varepsilon }}=m$$

m was the strain-rate sensitivity of the material. The power dissipation efficiency (η) was introduced to characterize the power dissipation during the evolution of the microstructure, and it was calculated using the following equation [25]:

(21) $$\eta =\frac{J}{J_{\max}}=\frac{2m}{m+1}$$

The variation of η with strain rate and deformation temperature constitutes the power dissipation diagram. This graph was a contour map of the variation of deformation temperature–strain rate field efficiency, with different regions representing specific deformation mechanisms. Furthermore, a criterion of flow instability was defined using the extremum principle of irreversible thermodynamics [36,40]:

(22) $$\xi (\dot{\varepsilon })=\frac{\partial \ln\left(m/m+1\right)}{\partial \ln\dot{\varepsilon }}+m\leq 0$$

**Figure 9:**
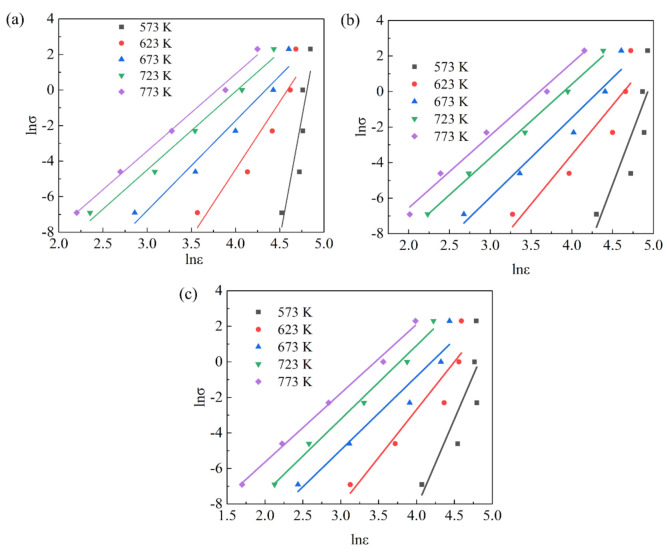
The relationship of lnσ-ln$\dot{\varepsilon }$, (**a**) ε = 0.1, (**b**) ε = 0.3, (**c**) ε = 0.5.

Flow instability occurred when the ξ($\dot{\varepsilon }$) was a negative value. According to the thermal compression curves of the Mg-2.5Nd-0.5Zn-0.5Zr alloy in Figure 2, the flow stress at different deformation temperatures (T), strain rates ($\dot{\varepsilon }$) and strain variables (ε) was obtained, and the relationship of the lnσ-ln$\dot{\varepsilon }$ at given strain conditions was plotted. Figure 9 shows the lnσ-ln$\dot{\varepsilon }$ at the strain variables of 0.1, 0.3 and 0.5. The lnσ and ln$\dot{\varepsilon }$ had good linear correlations. This indicated that the stress–strain of the Mg-2.5Nd-0.5Zn-0.5Zr alloy in the range of 573–773 K/0.001–10 s^−1^ followed the power equation of Equation (15). This showed that the DMM and Prasad criterion could be used to draw the processing maps of the Mg-2.5Nd-0.5Zn-0.5Zr alloy under different strain conditions.

**Figure 10:**
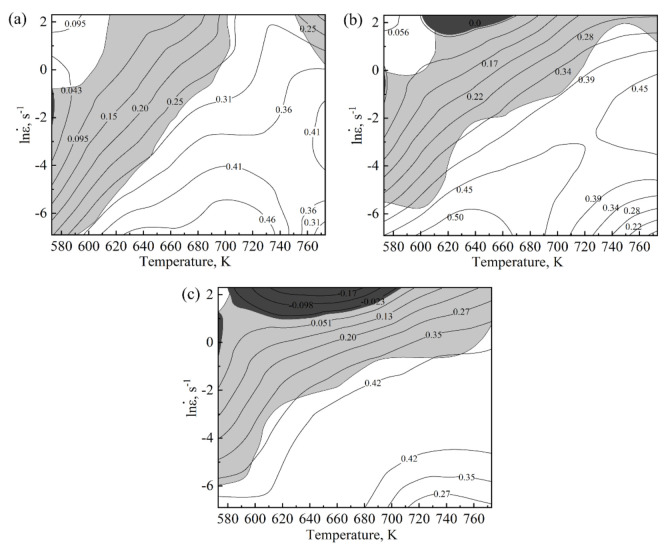
The processing map of the Mg-2.5Nd-0.5Zn-0.5Zr alloy, (**a**) ε = 0.1, (**b**) ε = 0.3, (**c**) ε = 0.5.

Figure 10 shows the processing maps and instability diagram which were superposed by power dissipation diagram and the stress–strain curve of the Mg-2.5Nd-0.5Zn-0.5Zr alloy at strain variables 0.1, 0.3 and 0.5, respectively. The value of the isoline in the figure represents the percentage of the power dissipation factor (η), which represents the energy consumed by the change of the tissue during thermal deformation. Generally, the value of η corresponding to DRV ranged from 20 to 30%, whereas that of DRX pairs was above 30%. In Figure 10, the gray shaded area represents the region where $\xi (\dot{\varepsilon })$ was less than 0, i.e., the instability zone of plastic flow. When ε was 0.1, η increased with increased temperature and decreased with strain rate in the range of 573~723 K. At 773 K, the value of η showed a decreased trend. When ε was 0.3 and 0.5, the variation law was essentially the same as when ε was 0.1. When ε was 0.1, the η of the instability zone was less than 30%. The instability zones were two discontinuous zones at 573–592 K and 0.001–0.555 s^−1^, and 759–773 K and 2.513–10 s^−1^. The stable region was at 592–773 K and 0.001–10 s^−1^. When the ε increased to 0.3, the η of the instability region reached 35%, and the instability region was 573–773 K and 0.003–1.176 s^−1^. The stable regions were 573–610 K and 0.001–0.003 s^−1^, 610–705 K and 0.003–0.441 s^−1^, 705–773 K and 0.411–9.081 s^−1^, respectively. When the strain increased to 0.5, the η of the instability region reached 42%, and the instability zone was 573–773 K and 0.002–4.758 s^−1^. The stable regions were 573–597 K and 0.001–0.004 s^−1^, 597–664 K and 0.004–0.181 s^−1^, 664–773 K and 0.181–1.416 s^−1^, respectively. In summary, the best processing area was 592–773 K and 0.001–0.217 s^−1^.

### 3.4. Microstructure Evolution of the Compression Alloy Was Analyzed by EBSD

**Figure 11:**
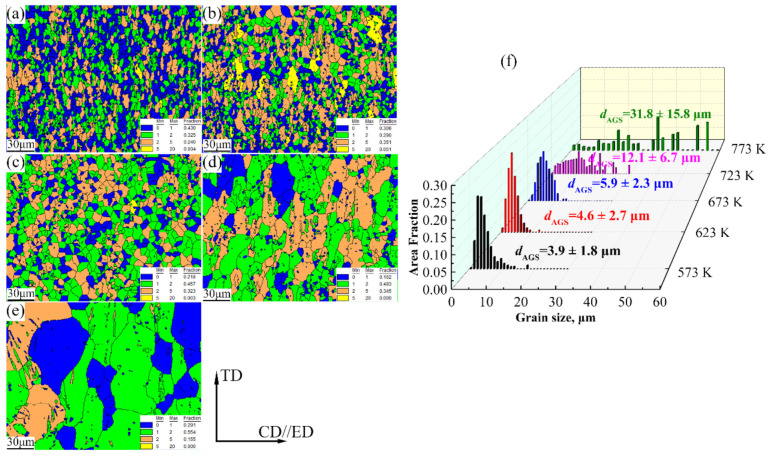
GOS maps of the Mg-2.5Nd-0.5Zn-0.5Zr alloy deformed at different temperatures and a strain rate of 0.001 s^−1^, (**a**) 573 K, (**b**) 623 K, (**c**) 673 K, (**d**) 723 K, (**e**) 773 K and (**f**) grain size distribution. (ED: extrusion direction, CD: compression direction, TD: transverse direction).

Figure 11 exhibits the EBSD figures and 3D grain size distribution diagrams of the Mg-2.5Nd-0.5Zn-0.5Zr alloy in various hot deformation conditions. In this study, the method for distinguishing recrystallized and deformed grains was that described in Ref. [3]. With the increase in deformation temperature, the grain size of the alloy gradually grew. The average grain sizes were 3.9 ± 1.8 μm (573 K), 4.6 ± 2.7 μm (623 K), 5.9 ± 2.3 μm (673 K), 12.1 ± 6.7 μm (723 K) and 31.8 ± 15.8 μm (773 K), respectively (Figure 11f). The grain size of the alloy grew slowly in the 573–673 K deformation temperature range. The grain size of the alloy grew rapidly in the 723–773 K deformation temperature range. The Zener–Hollomon ($Z=\dot{\varepsilon }\exp\left(-\frac{Q}{\mathrm{RT}}\right)$) parameters determine whether dynamic recrystallized grains were refined during plastic deformation [22]. With the increase in deformation temperature, the *Z* value was larger, which weakened the grain refining effect of DRX and resulted in coarse recrystallized grains [15]. Meanwhile, the grain boundary migration speed was accelerated, and grains noticeably grew [32]. The recrystallization proportions of the alloys were 75.5% (573 K), 59.8% (623 K), 67.5% (673 K), 65.5% (723 K) and 84.5% (773 K), respectively. Interestingly, the hot compression experiment was not a transient process, and new dynamically recrystallized grains were involved in deformation during nucleation and growth. Hence, the degree of DRX could only be explained qualitatively according to the information in the GOS figure, while the actual DRX was different from that described in the GOS figure [30,31,32,33,34,35,36,37,38,39,40,41].

**Figure 12:**
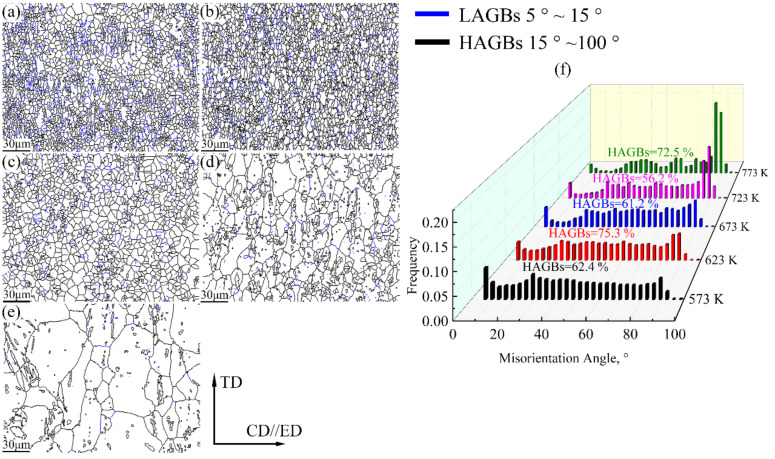
Grain boundary maps of the Mg-2.5Nd-0.5Zn-0.5Zr alloy deformed at different temperatures and a strain rate of 0.001 s ^−1^. Black lines refer to HAGBs (>15°), blue lines to LAGBs (5~15°), (**a**) 573 K, (**b**) 623 K, (**c**) 673 K, (**d**) 723 K, (**e**) 773 K and (**f**) Misorientation angle distribution.

Figure 12 presents the GB figures of the Mg-2.5Nd-0.5Zn-0.5Zr alloy at various hot deformations. During hot deformation, dislocations accumulated and rearranged at grain boundaries and severely deformed regions, forming low-angle grain boundaries (LAGBs) [2]. With the increase in deformation temperature, LAGBs were transformed into subgrains by absorbing surrounding dislocation, and the sub-grains grew to form high-angle grain boundaries (HAGBs). LAGBs continuously absorbed dislocations into HAGBs, resulting in a significant increase in HAGBs. Meanwhile, the increase in HAGBs meant that the stored dislocation was consumed, which was consistent with the characteristics of CDRX [30]. As shown in Figure 12, with the increase in deformation temperature, the proportion of HAGBs of the alloy increased first, then decreased and finally increased. When the deformation temperature was 773 K, the recrystallization ratio of the alloy was 84.5%, which indicates that the ability of LAGBs to absorb dislocation and transform into HAGBs was improved at high temperatures, providing a sufficient driving force for DRX [42]. This was consistent with the recrystallization trend of the alloy shown in Figure 11.

**Figure 13:**
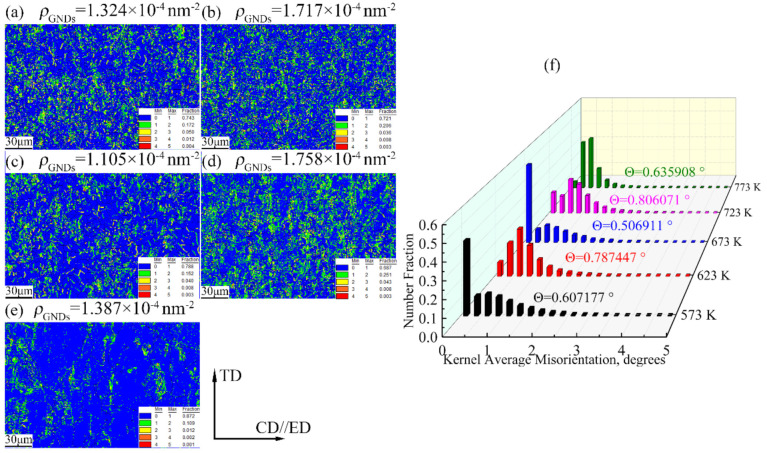
The kernel average misorientation of the Mg-2.5Nd-0.5Zn-0.5Zr alloy deformed at different temperatures and a strain rate of 0.001 s^−1^. (**a**) 573 K, (**b**) 623 K, (**c**) 673 K, (**d**) 723 K, (**e**) 773 K and (**f**) kernel average misorientation distribution.

Figure 13 depicts the KAM of the Mg-2.5Nd-0.5Zn-0.5Zr alloy at various hot deformations. The KAM diagram was calculated based on the local offset level between a single point (core) and all surrounding points and was usually used to represent the internal plastic strain of the alloy. The higher the KAM value was, the greater the plastic deformation degree was, and the greater the geometry had to be dislocated. The geometry dislocation calculation method was that described in Refs. [6,15]. The gradual change from blue to red in the KAM maps shows that the local directional deviation was gradually increasing. In Figure 13, it is shown that the KAM values of the alloy were 0.607177° (573 K), 0.787447° (623 K), 0.506911°(673 K), 0.806071° (723 K) and 0.635908° (773 K), respectively. The calculated geometry must have had dislocations of 1.324×10^−4^ nm^−2^ (573 K), 1.717 × 10^−4^ nm^−2^ (623 K), 1.105 × 10^−4^ nm^−2^ (673 K), 1.758 × 10^−4^ nm^−2^ (723 K) and 1.387 × 10^−4^ nm^−2^ (773 K), respectively. However, no matter what the deformation temperature was, the local misorientation was not uniform during hot compression, and the misorientation at the grain boundary was generally higher than that in the grain [30]. These results indicate that the dislocation accumulation at the grain boundary was severe and the deformation storage was large, which provided the energy for DRX nucleation. In addition, the atoms on the grain boundary were rearranged irregularly and the lattice distortion was relatively large, which made the absorption of various impurity elements easy. Therefore, in the process of plastic deformation, grain boundaries hinder dislocation movement, and dislocation stacking groups tend to appear in front of grain boundaries [30]. As shown in Figure 13, with the increase in deformation temperature, local deflection first increases, then decreases, then increases and then decreases. This indicates that DRX occurs at the cost of dislocation consumption during hot compression. When this is combined with Figure 11, it can be seen that when the deformation temperature of the alloy was 773 K, the recrystallization ratio was relatively high and dislocation consumption was relatively large. Therefore, the $\rho_{\mathrm{GND}}$ was relatively small.

## 4. Conclusions

In this paper, the high-temperature deformation behavior of the Mg-2.5Nd-0.5Zn-0.5Zr alloy in the range of 573–773 K and 0.001–10 s^−1^ was studied using an isothermal compression experiment system. The main results were as follows:

(1) The flow stress of the Mg-2.5Nd-0.5Zn-0.5Zr alloy in the range of 573–773 K/0.001–10 s^−1^ was very sensitive to the deformation temperature, strain rate and strain variables. The whole hot-working process was divided into three stages: work hardening, dynamic softening and dynamic balancing.

(2) A hyperbolic Arrhenius-type strain compensation constitutive model with high accuracy for the Mg-2.5Nd-0.5Zn-0.5Zr alloy was established. This constitutive model had high acceptable predictability. The dominant deformation mechanism of the Mg-2.5Nd-0.5Zn-0.5Zr alloy was dislocation climbing.

(3) The thermal processing maps of the Mg-2.5Nd-0.5Zn-0.5Zr alloy under different strains were established, and the optimal processing range was determined to be 592–773 K and 0.001–0.217 s^−1^.

(4) With the increase in deformation temperature, the grain size of the Mg-2.5Nd-0.5Zn-0.5Zr alloy grew slowly at the 573–673 K temperature range and grew rapidly at the 673–773 K temperature range.

Highlights:

1. Hot deformation was performed in compressive modes.
2. A manufacturability map of the Mg-2.5Nd-0.5Zn-0.5Zr alloy was established for the first time.
3. A strain-compensated constitutive model for determining flow stress in this alloy was established with highly acceptable predictability.
4. The dominant deformation mechanism of the alloy was dislocation climbing.

## Figures and Tables

**Table 1:** The coefficient of material constants and true strain during fitting polynomials.

Material Constants				**Coefficients**			
	$\varepsilon^{0}$	$\varepsilon^{1}$	$\varepsilon^{2}$	$\varepsilon^{3}$	$\varepsilon^{4}$	$\varepsilon^{5}$	$\varepsilon^{6}$
α	0.01	0.09	−0.65	2.51	−5.09	5.03	−1.93
n	15.56	−170.07	1127.68	−4034.49	7904.61	−7960.32	3217.49
Q	507.57	−5781.23	43757.45	−170718.30	354866.01	−373319.75	156209.15
lnA	88.95	−1033.31	7893.32	−31410.53	66790.89	−71967.43	30880.84

**Table 2:** Stress exponent values and associated deformation mechanisms.

**(** **n** **) Values**	**Deformation Mechanisms**	**Ref.**
2	Grain boundary sliding	[25]
3	Viscous glide of dislocation	[26]
5	Dislocation climb	[40]
8 and above	Cross-slip of screw dislocations/Constant substructure model	[36]

**Table 3:** The activation energy (Q) and stress exponent (n) of the alloy at a strain of 0.4.

Temperature (K)	Q (kJ/mol)					n
	0.001 s^−1^	0.01 s^−1^	0.1 s^−1^	1 s^−1^	10 s^−1^	
573	271.9	268.9	224.3	197.7	144.4	4.86
623	203.5	201.3	167.9	148.1	108.1	3.64
673	199.3	197.1	164.4	144.9	105.9	3.56
723	197.8	195.7	163.2	143.9	105. 0	3.54
773	182.7	180.7	150.7	132.8	97.1	3.27

## Data Availability

The data presented in this study are available on request from the corresponding author. The data are not publicly available due to their association with an ongoing study.
